# Cognitive Fitness Framework: Towards Assessing, Training and Augmenting Individual-Difference Factors Underpinning High-Performance Cognition

**DOI:** 10.3389/fnhum.2019.00466

**Published:** 2020-01-14

**Authors:** Eugene Aidman

**Affiliations:** ^1^Land Division, Defence Science & Technology Group, Edinburgh, SA, Australia; ^2^School of Psychology, The University of Sydney, Sydney, NSW, Australia; ^3^School of Biomedical Sciences & Pharmacy, University of Newcastle, Newcastle, NSW, Australia

**Keywords:** cognitive fitness, task performance, operational readiness cycle, RDoC domains cognitive functioning, measurement, trainability

## Abstract

The aim of this article is to introduce the concept of Cognitive Fitness (CF), identify its key ingredients underpinning both real-time task performance and career longevity in high-risk occupations, and to canvas a holistic framework for their assessment, training, and augmentation. CF as a capacity to deploy neurocognitive resources, knowledge and skills to meet the demands of operational task performance, is likely to be multi-faceted and differentially malleable. A taxonomy of CF constructs derived from Cognitive Readiness (CR) and Mental fitness (MF) literature maps into phases of operational cycles from foundational to advanced, mission-ready and recovery. Foundational cognitive attributes, such as attention, executive control and co-action, were hypothesized to be trainable at the initial Cognitive Gym phase. More advanced training targets at the CR phase included stress and arousal regulation, adaptability, teamwork, situation awareness (including detection, sense-making and prediction) and decision making (de-biasing and confidence calibration). The mission-ready training phase is focused on tolerances (to sleep loss, monotony, pain, frustration, uncertainty) and resistance (to distraction, deception or manipulation). Operational Augmentation phase relies on support tools such as decision aids and fatigue countermeasures, while the Recovery phase employs reflexive (e.g., mindfulness), and restorative practices (e.g., nutrition and sleep hygiene). The periodization of cognitive training in this cycle is hypothesized to optimize both real-time cognitive performance and the resilience that enables life-long thriving. One of the most promising avenues of validating this hypothesis is by developing an expert consensus on the key CF ingredients and their relative importance in high-performance settings.

## Introduction

Performance psychology is a rapidly expanding field that is of growing significance to a wide range of occupations—from competitive sport and performing arts to first responder and military professions. These user groups share a common focus on striving for superior performance in challenging tasks under stressful conditions, and on effective recovery to enable repeat performance across the lifespan. The physical and psychological factors contributing to task performance are tightly interconnected and go beyond mere “wellness” (i.e., the absence of pathology). They include, apart from knowledge and skills, a range of “capacity” factors, such as strength, endurance, and flexibility, that is best summarized by the concept of “fitness.” This article introduces the concept of Cognitive Fitness (CF) as a capacity to deploy neurocognitive resources underpinning the execution of goal-directed action and proposes a hypothetical set of its ingredients. Similar to physical fitness (PF), CF enables the application of knowledge, skills and attitudes (KSA) in generating task performance. The ingredients of PF are well established, with robust measurement protocols for muscular strength, aerobic/anaerobic endurance and range of motion/joint flexibility (Jeffreys and Moody, 2016), as well as validated training interventions such as strength and conditioning, cardiovascular fitness, or high-intensity interval training.

The meaning of *psychological fitness* is less clear, with this widely used term referring to diverse characteristics ranging from “character strengths and assets” (Cornum et al., 2011; Vie et al., 2016) to “resources that provide protection against the development of mental disorders” (Wesemann et al., 2018). The concept of *mental fitness* (MF) has emerged in the mental health and positive psychology literature (McCarthy, 1964; Seligman, 2008) to promote a positive and proactive notion of mental health. The MF literature is focused on identifying protective factors, such as cognitive flexibility, implicated both in the prevention of mental illness and in the promotion of flourishing (Keyes, 2007; Robinson et al., 2015). MF is also critical in the world of work, especially in high-stakes occupations where cognitive lapses can undermine the performance of complex socio-technical systems, while individual’s and teams’ superior capacity to sense, think, decide and act is widely seen as conferring critical performance advantages (Baker and Phillips, 2000; Bowers and Cannon-Bowers, 2014; Fletcher and Wind, 2014; Herzog and Deuster, 2014; Ahn and Cox, 2016; Bogga, 2017).

The growing literature on *cognitive readiness* (CR; Foster, 1996; Morrison and Fletcher, 2002; Grier, 2011; O’Neil et al., 2014) has developed extensive conceptual models of factors contributing to sustained professional performance in complex, dynamic, and unpredictable environments (Bolstad et al., 2006; Grier, 2012). In particular, CR has been construed as a comprehensive set of predictors—both distal and proximal—of cognitive performance by the military personnel in complex missions facing agile, near-peer opposition (Kluge and Burkolter, 2013; Sotos, 2019). The key components of CR include: (1) trainable skills, knowledge and attitudes (KSAs); (2) dynamic functional states; and (3) stable, trait-like characteristics ranging from cognitive ability to working memory and learning styles (Grier, 2012; Mason and McQuade, 2013). CF (Aidman, 2017) corresponds to the cognitive element of this latter CR component. Despite the lack of causal cognitive mechanisms explained by the CR construct (Crameri et al., 2019) it has been instrumental in stimulating the development of measurement frameworks for assessing individuals’ and teams’ fluctuating capacity for operational task performance (Fatkin and Patton, 2008; Grier et al., 2012) and evaluating training interventions to improve it (Kluge and Burkolter, 2013; Peña and Brody, 2016). The explanatory power of the CR construct can be enhanced by establishing its connections to causal factors, such as arousal regulation, discomfort tolerance and inhibitory control, underpinning an individual’s performance in cognitively demanding tasks. These fundamental, biologically traceable dimensions of cognitive functioning have been well established in clinical neuroscience (Cuthbert and Insel, 2013; Appelbaum, 2017). Their incremental predictive validity for the assessment of psychopathology (e.g., Yücel et al., 2019) and mental health in nonclinical populations (Carcone and Ruocco, 2017) indicates that these broad domains of cognitive functioning can potentially underpin CR as well. The concept of CF (Aidman, 2017) was introduced to examine these causal connections between CR and cognitive factors underpinning mental health, to bridge the gap between the CR and MF literature, and to develop a more tractable and systemic approach to the assessment and training of high-performance cognition. CF is focused on the “capacity” component of CR—as distinct from its “knowledge, skill and expertise” (KSE) components. The aim of the current article is to develop a working definition of CF, by drawing on the “why,” the “what” and the “how-to” questions from the cognitive training and readiness literatures, and connecting them to the broad, biologically traceable domains of cognitive functioning developed in clinical neuroscience. A hypothesized set of constituent elements of CF is then articulated in a Cognitive Fitness Framework (CF2), followed by a discussion of its potential applications in the areas of assessment, training periodization and operational augmentation. Finally, a research agenda is suggested to improve CF2 and the measurement tools supporting it.

## Cognitive Training: from “How To” to “What” and “Why”

MF has been defined as a set of malleable attributes that can develop through regular practice—analogous to physical training (Robinson et al., 2015). How trainable are the elements of CF is an empirical question. The growing literature on cognitive training has accumulated promising evidence of its effectiveness in areas such as visuospatial training in developmental populations (Boccia et al., 2017) and memory training for older adults (Gross et al., 2012; although see Redick, 2019; for a broader analysis of the latter).

More specifically, the occupational performance literature has accumulated promising evidence of the cognitive attributes that are considered both trainable and capable of producing reliable performance gains for the end-user—be it an athlete (Morris and Summers, 2004; Fadde and Zaichkowsky, 2018), police officer (Page et al., 2016), first-responder (Joyce et al., 2019), or a warfighter (Adler et al., 2015; Cooper and Fry, 2018; Blacker et al., 2019).

Despite the inconsistent evidence for the effectiveness of cognitive training (Walton et al., 2018; Redick, 2019) and substantial gaps in conceptual integration of cognitive attributes relevant to high-stakes performance applications, core psychological skills such as goal setting, imagery, attention and stress/arousal regulation have been shown to improve with systematic, deliberate practice (Zaichkowsky and Peterson, 2018) characterized by immediate performance feedback and gradual improvement through repetition (Ericsson, 2008). However, the literature is not clear on the parameters of such practice, what it should target and when—or even on how complete this skillset is. Where do you start? What should you train first, second, last? In what combinations, doses, frequency, with what recovery times? At what phase of your training cycle? If you have to choose, which ones are more important? What are the skill fade rates and refresher training requirements? This systemic picture seems missing here, with the resulting symptoms of slow progress, such as persistently low rates of transfer of cognitive training to untrained tasks (Redick, 2019). At the same time, the field is flooded with a plethora of technological inventions for cognitive training and augmentation that are more focused on demonstrating the diverse new technologies than on what cognitive faculties are worth training for (with the attendant questions about the limits of their trainability), and what the best methods of training them are in a holistic and pragmatic approach that accounts for how these faculties develop and fade, improve and decline through existing real-life processes of maturation, aging, training and education, medical treatment, et cetera.

Addressing the questions of trainability would open up a realistic, evidence-based consideration of those cognitive attributes that are either unlikely to improve through training (and thus should be selected for) or fluid/cyclical in nature (and thus need to be monitored and augmented). Unpacking the neurocognitive mechanisms underlying task performance is critical to assessing attribute trainability. This mechanistic analysis requires the increasingly relevant evidence from neurosciences about factors impacting cognitive functioning—from genetics to social interactions.

## Domains of Cognitive Functioning

With a ground-swell in the mental health literature suggesting that mental illness is not a category, CF2 suggests that neither is high-performance. Both are natural consequences of the varying levels of psychological functioning (including cognitive, affective and motivational) ranging from deficit to norm, and further to high or gifted performance. Broad expert consensus exists on key domains of cognitive functioning that underpin mental health (Morris and Cuthbert, 2012; Yücel et al., 2019). The deficiency of categorical diagnostic systems is well known—DSM-5 and ICD are being challenged by the Research Domain Criteria (RDoC; Cuthbert and Insel, 2013) framework with its broad dimensions of cognitive functioning (Appelbaum, 2017; Clark et al., 2017).

RDoC defines “major domains for the study of mental illness and validate them using optimal genetic, neuroscientific, physiological, behavioral, and self-report measures” (Morris and Cuthbert, 2012). The long-term goals of RDoC were to validate tasks for use in clinical trials, identify new targets for treatment development, and provide a pathway by which research findings can be translated into changes in clinical decision making. RDoC identified broad higher-level domains of functioning that comprise multiple sub-dimensional constructs, reflecting state-of-the-art knowledge about major systems of cognition, motivation, and social behavior. In its present form, the RDoC Matrix contains five broad Domains cognitive functioning that are differentiated into 23 main Constructs (shown in brackets here) which are further divided into Subconstructs (Clark et al., 2017):

1. Negative Valence Systems (fear, anxiety, sustained threat, loss, frustration).
2. Positive Valence Systems (approach motivation, initial and sustained responsiveness to reward, reward learning, and habit).
3. Cognitive Systems (attention, perception, working and declarative memory, language, and cognitive control).
4. Social Processing Systems (affiliation/attachment, communication, self/other-perception).
5. Regulatory Systems (arousal, circadian rhythms, sleep and wakefulness).

The full list of RDoC constructs is regularly updated at https://www.nimh.nih.gov/research/research-funded-by-nimh/rdoc/constructs/rdoc-matrix.shtml. The constructs are being continuously revised and refined, with the overall goal of improving measurement validity and treatment efficacy. Expert consensus frameworks have become a best-practice standard, they are known to stimulate research discoveries and accelerate translational pathways by estimating the relevance of primary RDoC constructs (and their sub-dimensions) to specific application domains such as substance and behavioral addictions (Yücel et al., 2019).

RDoC has informed the development of reliable and valid measures across a range of units of analysis for each construct—from genes and cells to neurocircuits, whole-body physiology (e.g., heart-rate or event-related potentials), behavior and subjective experience (e.g., Passell et al., 2019). These measures have enabled and inspired studies to determine the full range of variation along with these measurement constructs, from deficit to norm and characterizing both clinical and nonclinical populations (Carcone and Ruocco, 2017). Extending this range to the well-adjusted functioning and high-performance domains is an important next step, given that nonclinical populations have been under-represented in the current RDoC-driven research. This would require developing an expert consensus on the relative importance of primary RDoC constructs and their sub-dimensions to various high-performance applications. Consequently, CF2 is aimed at building on RDoC foundational evidence in order to define major domains for the study of CF and develop guidelines for assessing them using an optimal mix of biomarker, physiological, behavioral, and self-report measures (Aidman, 2017).

## Cognitive Fitness Framework

Integrating the evidence about the mechanisms of cognitive deficit and psychopathology (the RDoC literature) with what is relevant to high performance by well-adjusted individuals who are motivated to excel, remains incomplete. While the exact composition of RDoC domains relevant to work performance is awaiting full articulation through consensus studies similar to Robinson et al. (2015) and Yücel et al. (2019), their preliminary scoping can be informed by the widely recognized “psychological skillset” established in performance psychology (Zaichkowsky and Peterson, 2018). In exercise sciences, the ingredients of PF are well-established and include strength/power, endurance, agility and flexibility (Jeffreys and Moody, 2016). The corresponding features of cognitive performance include focus intensity (Sherlin et al., 2013) for strength, attention span and mental effort tolerance (Aidman et al., 2002, 2016; Aidman, 2005) for endurance, task shifting (Genet and Siemer, 2011), cognitive flexibility and creativity (Palmiero et al., 2019) for flexibility, and adaptability (Chandra and Leong, 2016; Zhang et al., 2019) and self-regulation (Schunk and Greene, 2017) for agility. Research evidence accumulated in sport psychology and other high-performance contexts, points to the same core domains of cognitive functioning, while their relative importance may depend on the specifics of task and mission profiles under consideration.

In particular, the MF resource index (Robinson et al., 2015) populates the same three categories—strength, endurance, and flexibility—with a set of positive psychology constructs such as self-efficacy (for strength), acceptance (for flexibility) and resilience (for endurance). These allocations are metaphorical—they “employ metaphor” (Robinson et al., 2015, p. 56) to create constructs that are similar to the well-understood components of PF. As a result, their neuro-psychological bases remain unclear, and the question of their measurement is left wide open. The extensive set of CR constructs (Cosenzo et al., 2007; Grier, 2011, 2012; O’Neil et al., 2014) is focused on higher-order abilities, such as decision making, problem-solving and metacognition, it also contains some underlying cognitive capacity constructs of agility, speed of processing and memory capacity (for review, see Crameri et al., 2019).

Based on the literature summarized above, CF can be defined as a “multi-faceted and differentially malleable capacity to deploy neurocognitive resources, knowledge, and skills to meet the demands of operational task performance, and to sustain this performance throughout a career- and life-long application.”

The key aspects of this working definition are:

1. CF entails multiple capacity factors that are different from knowledge and skills.
2. Operational task performance is core to defining the composition and relative importance of these factors.
3. The malleability of individual CF factors is to be established empirically.
4. Sustained performance implies resilience, well-being and longevity as by-products of fitness.

Several factors impacting task performance and career longevity in high stakes occupations can be considered for inclusion in the CF set. Table 1 summaries these operationally relevant constructs and notionally allocates them to the phases of the operational readiness cycle: from the foundational (cognitive gym) to advanced (readiness), mission-ready (operational tolerances), and recovery phase.

**Table 1:** Phases of cognitive fitness (CF) cycle: from training to operational performance and recovery.

Phase	The domain of cognitive functioning	Target constructs	Examples of training/development objectives^a^
Cognitive gym: Foundational training	Cognitive fitness:	Self-awareness	Stress symptoms detection
	Trainable cognitive primaries	Attention	Focus endurance
		Task switching	Dual-tasking
		Impulse control	Response override
		Co-action	Action mirroring
			Handover execution
Advanced cognitive training	Cognitive skills	Controlled response	Effortless concentration
		Energy management	Arousal regulation
			Progressive muscle relaxation
			Resonant frequency breathing
		Situation awareness	Perceptual acuity (detection)
			Sense-making (interpretation)
			Anticipatory skills (prediction)
		Decision making	Pattern recognition
			Bias detection and mitigation
			Confidence calibration
		Adaptability	Change detection
			Cognitive flexibility
		Teamwork	Shared mental models
			Non-verbal communication
Mission-ready training	Tolerance and resistance	Tolerances	Generalized discomfort tolerance
			Pain tolerance
			Alertness upregulation (drowsiness tolerance)
			Mental effort tolerance
			Monotony tolerance
			Frustration tolerance
			Ambiguity tolerance
			Startle/surprise tolerance
		Resistances	Distractor resistance
			Susceptibility to deception
			Resistanceto manipulation
		Task resilience	Error detection
			Performance recovery∣rule
Operational augmentation	Operational task performance	Cognitive state	Alertness monitoring^b^
		Cognitive workload	Fatigue countermeasures^b^
		Decision making	Adaptive decision aids^b^
		Equipment use	Operator state-aware autonomy^b^
Recovery	Cognitive recovery	Sleep recovery	Sleep/wake cycle management
		Reflexive practices	Mindfulness and meditation
		Nutrition	Healthy eating habits^c^
			Hydration^c^
		Connectedness	Interactional competence
			Social support^c^

*Notes: ^a^The list is not exhaustive and is subject to validation through expert consensus. These training objectives are not directly linked to operational task performance. Similar to strength and conditioning in physical training, the products of CF training feed into the subsequent cognitive skills training, and only through this skill training—into operational performance. ^b^Given the nature of the Operational Augmentation phase, performance augmentation tools are listed here instead of training objectives. ^c^The recovery phase is focused on the development of habits and practices that promote cognitive fitness and, as such, they are applicable to all other phases of the Cognitive Fitness Cycle*.

In particular, cognitive primaries, such as *attention*, *executive control*, and *co-action*, that underpin most cognitive skills, may potentially be trainable through the gold-standard “isolate—overload—recover” regimes at the initial, Cognitive Gym phase (Temby et al., 2015). More advanced training targets at the CR phase include *stress management* (arousal regulation and “getting in The Zone on cue”), *adaptability*, *teamwork*, *situation awareness* (including detection, sense-making and prediction) and *decision making* (de-biasing and confidence calibration). The mission-ready training phase is focused on *tolerances* (from pain and sleep loss to monotony, frustration and uncertainty) and *resistances* (to distraction, deception or manipulation). These capacities get further enhanced through operational support (with tools such as *decision aids* and *fatigue countermeasures*). The recovery phase completes the cycle, with its role widely recognized by expert consensus (Kellmann et al., 2018; Reardon et al., 2019), employing both reflexive (e.g., mindfulness) and restorative practices (e.g., healthy eating, hydration and sleep hygiene) and relying on social support. The full cycle reinforces the cognitive fundamentals that are known for their contribution to both real-time cognitive performance under challenging conditions (Blank et al., 2014; Crameri et al., 2019) and the resilience that enables career longevity and life-long thriving (Seligman, 2008; Cornum et al., 2011).

## Periodization of the Cognitive Fitness Cycle

**Figure 1:**
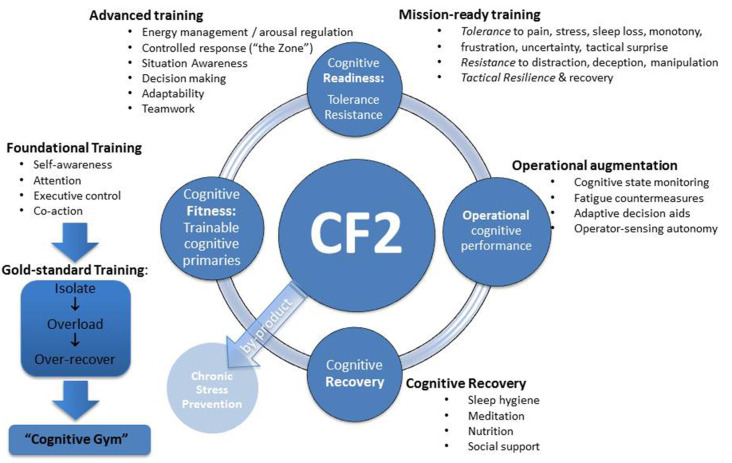
Cognitive fitness framework (CF2).

Some of the most important questions about the CF are centered around the sequential periodization of cognitive training. Figure 1 shows a hypothesized sequence in which these elements can be addressed in the training cycle across the CF2. The following questions seem worth addressing in future research:

1. What are the pattern-forming factors of CF? The factors that can drive change (both positive and negative) in multiple other factors. They should be trained first and foremost.
2. On what basis can training of some CF constructs be prioritized over the others?
3. What are the design principles for periodization of CF training to distinct micro-, meso- and macro-cycles to address the daily, weekly, monthly and yearly timescales (e.g., DeWiggins et al., 2010)?
4. How long is the optimal training cycle for a given CF attribute? Or how can this cycle be tailored for an individual?
5. Points of insertion into a holistic training cycle—the following options can be considered:

(a) CF training can be combined with existing training modalities that address physical fitness, technical skill acquisition or tactical skill application (Ward et al., 2017).
(b) CF training can be combined with mental/psychological skills training—the traditional province of sport psychology where self-management skills get added or overlaid to the already acquired fitness, technical and tactical skills (Birrer and Morgan, 2010).
(c) CF training can be a stand-alone modality—a largely neglected area where cognitive capacity training becomes part of the foundational holistic fitness.

It is safe to assume that the selection of insertion points will be guided by the task, context and resources available. In addition, their relative effectiveness can be compared directly (Röthlin et al., 2016).

## Applications

The CF2 is a step towards a consensus on the selection of cognitive attributes to train, the limits of their trainability, and the methods of assessing them in the 21st-century workforce. It helps to map out various lines of research effort and see where individual projects fit. For example, current research on foundational cognitive training is progressing under the construct of Cognitive Gym (Temby et al., 2015; Jarvis et al., 2019). On the other hand, the emerging work on team decision making has been driven by research evidence on the so-called *c*-factor (collective intelligence, or team “smarts”). This *c-factor* has surprisingly little connection to the individual team members IQ, and instead has been linked to their interactional competence and team diversity (Woolley et al., 2010; Blanchard et al., 2018).

One of the core predictors of fitness and performance is executive functioning (EF)—a primary cognitive capacity underpinning self-discipline, attentional focus and impulse control. Its known predictive links include BMI and cardiovascular health (Schlam et al., 2013), learning outcomes in academic and occupational training settings, injury incidence and overtraining, job performance and resilience, post-traumatic stress and other mental health vulnerabilities (Moffitt et al., 2011). Even a modest gain in EF capacity (either through selection or training) is known to drive population-wide gains in health, learning achievement in education and training (Moffitt et al., 2013), and productivity/work safety (including injuries). Twin studies indicate heavy genetic influences on EF (for review, see Friedman and Miyake, 2017), while longitudinal research shows substantial intra-individual variability (Moffitt et al., 2011). Direct estimation of EF heritability (vs. trainability) would go a long way towards informing wide-ranging investment decisions about selection and training programs. Adding this cognitive mediator to the original predictor set would make both performance and health prediction models more comprehensive and holistic.

## Conclusions and Future Directions

The construct of CF developed here is a means to address the gap between the CR, RDoC and MF literatures by offering a unifying framework to integrate the multitude of biologically traceable factors underpinning individuals’ performance in cognitively demanding tasks, to assess their trainability and inform the development of methods to improve them through training and augmentation.

The CF2 is a working hypothesis mapping out the research agenda to identify and measure key attributes of CF, underpinning both real-time cognitive performance under challenging conditions and the resilience that enables career longevity and life-long thriving. CF2 also offers a hypothesized sequence for cognitive training in a CF training cycle. As a hypothesis, CF2 requires testing and validation. One of the most promising validation avenues is through the development of an expert consensus on the key CF ingredients and their relative importance in high-performance settings. The Delphi method utilized in expert consensus studies of cognitive functioning in mental health (see Yücel et al., 2019) appears a good fit for validating the CF2 hypothesis. Once the relative importance of CF constructs is confirmed through expert consensus, training protocol evaluation studies can inform the selection of training methods that are best suited for each CF construct, including the formulation of training objectives to complement the training targets for each training phase in CF2.

The next challenge is extending the range of measurement of the assessment tools measuring CF constructs to cover both cognitive deficit and gifted performance and to employ best-practice measurement protocols to improve the reliability, validity and utility of these assessment tools. These improvements in the measurement of CF constructs are critical to stimulating the design and development of the environments and protocols to improve CF, and to developing fieldable technologies to protect and enhance cognitive performance.

## Data Availability Statement

No datasets were generated or analyzed for this study.

## Author Contributions

EA developed the conceptual framework for the article, drafted, revised, and approved the manuscript.

## Conflict of Interest

The author declares that the research was conducted in the absence of any commercial or financial relationships that could be construed as a potential conflict of interest.
