# Erratum: Cognitive Fitness Framework: Towards Assessing, Training and Augmenting Individual-Difference Factors Underpinning High-Performance Cognition

**DOI:** 10.3389/fnhum.2021.749619

**Published:** 2021-09-01

**Authors:** Frontiers Production Office

**Affiliations:** Frontiers Media SA, Lausanne, Switzerland

**Keywords:** cognitive fitness, task performance, operational readiness cycle, RDoC domains cognitive functioning, measurement, trainability

Due to a production error, there was an error in the reference details for Palmiero et al., 2019.

Instead of “Palmiero, M., Giulianella, L., Guariglia, P., Boccia, M., D'Amico, S., and Piccardi, L. (2019). The dancers' visuospatial body map explains their enhanced divergence in the production of motor forms: evidence in the early development. *Front. Psychol*. 10:1274. 10.3389/fpsyg.2019.01274,” it should be “Palmiero, M., Giulianella, L., Guariglia, P., Boccia, M., D'Amico, S., and Piccardi, L. (2019). The dancers' visuospatial body map explains their enhanced divergence in the production of motor forms: evidence in the early development. *Front. Psychol*. 10:768. 10.3389/fpsyg.2019.00768.”

The publisher apologizes for this mistake. The original article has been updated.

